# Humanization of the antigen-recognition domain does not impinge on the antigen-binding, cytokine secretion, and antitumor reactivity of humanized nanobody-based CD19-redirected CAR-T cells

**DOI:** 10.1186/s12967-024-05461-8

**Published:** 2024-07-25

**Authors:** Pooria Safarzadeh Kozani, Pouya Safarzadeh Kozani, Fatemeh Rahbarizadeh

**Affiliations:** 1 Department of Medical Biotechnology, Faculty of Medical Sciences, Tarbiat Modares University, Tehran, Iran; 2 Research and Development Center of Biotechnology, Tarbiat Modares University, Tehran, Iran

**Keywords:** Cancer immunotherapy, CD19, Nanobody, Humanization, Chimeric antigen receptor, Monoclonal antibody

## Abstract

**Background:**

The immunogenicity of the antigen-recognition domains of chimeric antigen receptor (CAR)-T cells leads to immune responses that may compromise the antitumor effects of the adoptively transferred T cells. Herein, we attempt to humanize a CD19-specific VHH (named H85) using in silico techniques and investigate the impact of antigen-recognition domain humanization on CAR expression and density, cytokine secretion, and cytolytic reactivity of CAR-T cells based on the humanized VHH.

**Methods:**

H85 was humanized (named HuH85), and then HuH85 was compared with H85 in terms of conformational structure, physicochemical properties, antigenicity and immunogenicity, solubility, flexibility, stability, and CD19-binding capacity using in silico techniques. Next, H85CAR-T cells and HuH85CAR-T cells were developed and CAR expression and surface density were assessed via flow cytometry. Ultimately, the antitumor reactivity and secreted levels of IFN-γ, IL-2, and TNF-α were assessed following the co-cultivation of the CAR-T cells with Ramos, Namalwa, and K562 cells.

**Results:**

In silico findings demonstrated no negative impacts on HuH85 as a result of humanization. Ultimately, H85CAR and HuH85CAR could be surface-expressed on transduced T cells at comparable levels as assessed via mean fluorescence intensity. Moreover, H85CAR-T cells and HuH85CAR-T cells mediated comparable antitumor effects against Ramos and Namalwa cells and secreted comparable levels of IFN-γ, IL-2, and TNF-α following co-cultivation.

**Conclusion:**

HuH85 can be used to develop immunotherapeutics against CD19-associated hematologic malignancies. Moreover, HuH85CAR-T cells must be further investigated in vitro and in preclinical xenograft models of CD19+ leukemias and lymphomas before advancing into clinical trials.

**Supplementary Information:**

The online version contains supplementary material available at 10.1186/s12967-024-05461-8.

## Introduction

The maintenance of CD19 expression in B-lineage cells even after their neoplastic transformation accentuates the importance of this molecule for the diagnosis and treatment of B-cell leukemias and lymphomas [1]. CD19-targeted cancer immunotherapies including monoclonal antibodies (mAbs; such as *tafasitamab*), T-cell-redirecting bispecific antibodies (TRBAs; such as *blinatumomab*), antibody-drug conjugates (ADCs; such as *loncastuximab tesirine*), and chimeric antigen receptor (CAR) T cells have created different trajectories in the fight against CD19-associated oncological indications [2–4]. CARs are synthetic receptors armed with an antigen-recognition domain fused to the singling domains necessary for driving the activation, expansion, and cytotoxicity effects of genetically manipulated T cells. Such antigen-recognition domains are mainly based on a single-chain fragment variable (scFv) derived from a mAb or a single variable domain of a camelid heavy-chain-only antibody known as a “VHH” or “nanobody^®^” [5, 6]. The surface expression of CARs on engineered T cells enables them to specifically target and eliminate cancer cells of interest in a major histocompatibility complex (MHC)-independent fashion. In 2017, the US Food and Drug Administration (FDA) granted *tisagenlecleucel* and *axicabtagene ciloleucel* clinical approval for the treatment of certain patients with conventional treatment-resistant relapsed/refractory (R/R) B-cell acute lymphoblastic leukemia (B-ALL) and diffuse large B-cell lymphoma (DLBCL), respectively. Later on, *brexucabtagene autoleucel* and *lisocabtagene maraleucel* were approved by the US FDA as treatments for certain patients with mantle cell lymphoma (MCL) and DLBCL, respectively. The approval of these four CD19-redirected CAR-T cells further highlights the clinical importance of CD19 in the context of cancer immunotherapy alongside accentuating the potential therapeutic benefits of CAR-T cells for the treatment of CD19-associated malignancies. Despite the tremendous clinical success of CAR-T cells, there have been some reports on the development of anaphylaxis in recipients caused by the production of neutralizing antibodies, most likely IgE, against the animal-derived antigen-recognition domains of CAR constructs [7, 8]. Such occurrences highlight the potential immunogenicity of the antigen-recognition domain of CARs derived from murine or camelid antibodies which might result in the incapacitation of the antitumor effects of CAR-T cells if not taken into consideration [9]. On the one hand, the utilization of fully human antigen-recognition domains in the construction of CARs can be considered to ameliorate these effects. On the other hand, since the application of fully human mAb-derived antigen-recognition domains with a desired range of affinity may not always be feasible, humanization of the animal antibody-derived antigen-recognition domains via the replacement of the animal frameworks with those of a human antibody might be a more considerate option.

In our previous study, we demonstrated that VHH-based CD19-redirected CAR-T cells (with a high-affinity CD19-specific VHH as the antigen-recognition domain) are capable of mediating robust antitumor effects comparable to their FMC63-based counterparts [6]. Of note, this CD19-specific VHH was selected and characterized in our other previous study from an immune camelid library using the phage display technique [10]. Herein, this CD19-specific VHH was humanized using in-depth and meticulous in silico techniques. Next, VHH-based CD19-redirected CAR-T cells based on the native or humanized VHHs were developed and assessed in terms of CAR expression rate and CAR surface density alongside antitumor effects and cytokine secretion (namely, IFN-γ, IL-2, and TNF-α) following their co-cultivation with target cells.

## Materials and methods

### In silico experiments

#### Humanization

In our previous study, an immune camelid VHH library was constructed and several CD19-specific VHHs were isolated from this library using the phage display technique [10]. From these VHHs, a high-affinity CD19-specific VHH with a high rate of specificity and sensitivity (termed H85) was selected as the antigen-recognition domain of CAR-T cells [10]. The hypervariable region of the heavy chain (V_H_) of a human antibody with high amino acid similarity to H85 was used for carrying out the humanization process. For this aim, a FASTA search was carried out to find the best match out of a vast repertoire of human antibody sequences registered in the Protein Data Bank (PDB). The Paratome server (http://www.ofranlab.org/paratome/) was employed for determining the complementarity-determining regions (CDRs) of H85 [11, 12]. The framework regions of the selected human antibody were used as the template for grafting the CDRs of H85 to them. This process results in the humanization of H85 (hereinafter HuH85) without amino acid manipulations of the native CDRs. All residue numberings have been carried out according to the *Kabat* numbering scheme [13].

#### 3D structure prediction and quality assessments

Several servers were utilized for predicting the 3D structures of H85 and HuH85 which included the Robetta server (https://robetta.bakerlab.org/), GalaxyWEB server (http://galaxy.seoklab.org/), and the Zhang server’s I-TASSER (https://zhanglab.ccmb.med.umich.edu/I-TASSER/) [14–18]. The quality of each of the predicted 3D structures was assessed using QMEANDisCo (https://swissmodel.expasy.org/qmean/) and ProSa (https://prosa.services.came.sbg.ac.at/prosa.php) [19–21]. Further vast analyses were also carried out using MolProbity (http://molprobity.biochem.duke.edu/) which included the Ramachandran plot analysis of each of the predicted structures and various other meticulous geometry analyses [22, 23]. UCSF Chimera software (version 1.17.3; CA, United States) was utilized for the root-mean-square deviation (RMSD) calculations between the aligned VHH structures [24]. All visualizations have been carried out using the PyMOL Molecular Graphics System (version 2.3.2; Schrödinger, LLC, United States).

#### Energy minimization

Energy minimization of a given predicted 3D model can help relieve severe clashes that occur during the process of modeling by the server. The energy minimization process of H85 and HuH85 was carried out using a two-step atomic-level energy minimization performed by the ModRefiner server (http://zhanglab.ccmb.med.umich.edu/ModRefiner/) [25]. This step ensures that any resultant predicted model used for the further steps of our experiment is free of any unfavorable clashes and in a stable and energetically favorable state.

#### Characterization

The ProtParam server (http://web.expasy.org/protparam/) was utilized to determine the physicochemical properties of H85 and HuH85 [26]. The server of ccSol (http://service.tartaglialab.com/new_submission/ccsol) was utilized to predict the solubility profile based on the physicochemical properties. Furthermore, ccSol omics (http://service.tartaglialab.com/new_submission/ccsol_omics), which uses mutational analysis and *E. coli* expression, was also employed to predict the solubility of the VHH before and after humanization. Moreover, the Aggrescan3D 2.0 server (http://biocomp.chem.uw.edu.pl/A3D2/) was used for the prediction of aggregation propensity to investigate whether the humanization process could unfavorably impact HuH85 [27]. Of note, in the results of the Aggrescan3D server, the average value is an indicator of the aggregation propensity of the protein structure, and more negative values are indicators of higher normalized solubility (with the threshold of solubility set at 0). The antigenicity of H85 and HuH85 was predicted using VaxiJen (http://www.ddg-pharmfac.net/vaxijen/VaxiJen/VaxiJen.html) to further validate the success rate of the humanization process [28, 29]. Additionally, the Immune Epitope Data Base’s (IEDB) Bepipred linear epitope prediction tool (http://tools.iedb.org/bcell/) was also employed for further immunological analyses [30]. Tm Predictor (http://tm.life.nthu.edu.tw/index.htm) was also used to theoretically calculate the protein melting temperature of the native and humanized VHH.

#### Flexibility, thermal stability, and surface accessibility assessments

The CABSflex server (http://biocomp.chem.uw.edu.pl/CABSflex2) was utilized for a fast simulation of protein structure flexibility to investigate whether amino acid substitutions following humanization could have substantial effects on the structural conformation of HuH85 as compared with that of H85 [31]. For this aim, the 3D structures of H85 and HuH85 were given to the server as input for the flexibility simulation process. Moreover, iStable 2.0 (http://ncblab.nchu.edu.tw/istable2/seqsubmit.html) was employed to predict the exact impact of each framework residue substitution on the thermal stability of the humanized VHH [32]. This step enables us to determine whether the humanized VHH is as thermally stable as its native counterpart. Furthermore, the surface accessibility of H85 and HuH85 was also computed by iStable 2.0 to further analyze the impact of humanization.

#### Molecular dynamics (MD) analysis

To investigate the stability of the native and humanized VHH, MD analysis was carried out. In this method, the protein atoms are placed at a specific temperature and pressure and then they are allowed to find the best locations once the force fields are applied. The stability of the native and humanized VHH is confirmed if they manage to preserve their native conformational structure over the course of the MD simulation and after the MD process has successfully finished. The MD simulation process was performed using the GROMACS software 2020 package and OPLS-AA force field [33, 34]. Each VHH was placed in the center of a cubic box, which was subsequently solvated with water molecules using the TIP3 model, with a distance of 1 nm from all edges [35]. Sodium (Na^+^) or chloride (Cl^−^) ions were used for the system neutralization whenever required. For the energy minimization of each system, the steepest decent algorithm was used. Subsequently, 100 picoseconds (ps) of NVT (N, constant number of particles; V, volume; T, temperature) and 100 ps of NPT (N, constant number of particles; P, pressure; T, temperature) were performed for the equilibration of each system. The RMSD and radius of gyration (R_g_) were the factors measured for the confirmation of VHH stability after running the MD simulation for 30 nanoseconds (ns). Also, the flexibility of each VHH per residue was measured over the course of the MD simulation.

#### CD19-binding efficacy assessments

For the docking of H85 and HuH85 to CD19, the ClusPro server (https://cluspro.bu.edu/login.php) was employed using the antibody mode and automatic masking of the non-CDR regions option offered by the server [36]. The most favorable docking complex was then used for the identification of the interactive residues in the binding complex of H85 or HuH85 to CD19 using the LigPlot^+^ software (version 2.2.8; Roman Laskowski) [37, 38]. Next, the PRODIGY server (https://wenmr.science.uu.nl/prodigy) was used for the prediction of the ΔG and dissociation constant (K_d_) of the docked complex of H85 to CD19 and HuH85 to CD19 [39, 40]. Of note, lower energy states are indicative of more stable and energetically favorable binding reactions. Additionally, the mCSM-PPI2 server (http://biosig.unimelb.edu.au/mcsm_ppi2/) was also employed to further analyze the impact of the framework residue substitutions on the affinity of the humanized VHH to CD19 [41].

### In vitro experiments

#### CAR cassette construction

The CD19-redirected CAR cassette was constructed with the CD19-specific VHH as the antigen-recognition domain, CD8α as the spacer domain, and 4-1BB and CD3ζ fragments as the intracellular signaling domains. H85CAR and HuH85CAR harbored H85 and HuH85 as their antigen-recognition domain, respectively. For the generation of CAR-T cells, primary T cells were transduced with third-generation lentiviruses. Briefly, two packaging plasmids (namely, pMDLg/pRRE and pRSV/Rev), one envelope plasmid (namely, pMD2G), and the pLJM1 transfer plasmid (PLJM1-EGFP; catalog number #19319; Addgene, MA, United States) were utilized for the encapsulation of the third-generation lentiviruses in this study.

Two distinct versions of the transfer plasmid were developed and used in the co-transfection process for the encapsulation of different CAR-integrating lentiviral particles. At first, the PLJM1-EGFP vector was digested at 5’ with EcoRI (Thermo Fisher Scientific, United States) and at 3’ with NdeI (New England Biolabs, United States) to insert a pre-digested DNA fragment for the EF1α promoter and the coding sequence of the H85CAR into the vector, as performed previously in our laboratory; this vector is hereinafter referred to as pLJM1-H85CAR. pLJM1-H85CAR is designed in a way that the antigen-recognition domain could be replaced with any given targeting moiety upon the digestion of the vector and insert fragment with NheI (New England Biolabs, United States) at 5’ and BstbI (New England Biolabs, United States) at 3’. The coding sequence of HuH85 was codon optimized and then synthesized by GenScript (GenScript Biotech, Piscataway, New Jersey, United States) in pUC57 (cat No. SD1176; GenScript Biotech, Piscataway, New Jersey, United States). This coding sequence was later PCR-amplified using the forward primer 5’-CTTtGCTAGCCAGGTTCAGCTGCAGGAATC-3’ and the reverse primer 5’-agacTTCGAAGGAGCCAGAAGAAACGGTAACCAGG-3’ which were designed to introduce the NheI and BstbI restriction sites at the 5’ and 3’ of the DNA fragment, respectively. The amplicons were then electrophoresed on 1% agarose gel and extracted from the gel using a commercial kit (Agarose Gel DNA Extraction Kit; Roche, Merck KGaA, Germany) as per the manufacturer’s protocols. Ultimately, the amplicons were digested with the NheI and BstbI enzymes and then ligated into the predigested pLJM1-H85CAR plasmid (from which the coding sequence of H85 had been digested out) only to develop pLJM1-HuH85CAR. This step was verified through DNA sequencing and enzymatic digestion using KpnI (New England Biolabs, United States), upon which 7171 and 2113 bp DNA fragments would be observed on 1% agarose gel.

#### Production of lentiviruses

Human embryonic kidney 293LTV cells (HEK 293LTV; Cell Biolabs, Inc., San Diego, CA, United States) were cultured in Dulbecco’s Modified Eagles Medium (DMEM; Gibco, Life Technologies, United States), and were employed for the production of different lentiviral particles. All culture media were supplemented (hereafter referred to as complete media) with 4 mM L-glutamine, penicillin and streptomycin (100 I.U./mL and 100 µg/mL; Gibco, Scotland, United Kingdom), 1 mM sodium pyruvate, and 10% fetal bovine serum (FBS; Gibco, Life Technologies, United States), and cells were cultivated in a humidified incubator with 5% CO_2_ at 37 °C.

For lentivirus production, 293LTV were cultured in 10 cm cell culture plates until reaching a confluency rate of 75–80%. Next, the two packaging, one envelope, and each transfer plasmid were mixed (at a ratio of (2:2):4:1, respectively) with polyethyleneimine (PEI; Sigma-Aldrich; Merck KGaA, Germany) at a ratio of 2.5:1 PEI:DNA to prepare a polyplex solution for the co-transfection step. The expression of GFP by 293LTV (for the transfection of which the PLJM1-EGFP transfer plasmid was used) was assessed under a fluorescence microscope 24, 48, 72, and 96 h following transfection to validate the process of lentivirus production. The culture medium containing the produced lentiviruses was collected 24, 48, 72, and 96 h after co-transfection. Next, the media was centrifuged at 1200 × g for 10 min to remove any cell debris, and the supernatant was collected and then filtered using 0.45 μm syringe filters (Sartorius, Goettingen, Germany). In the following, the lentiviruses were concentrated by centrifugation at 40,000 × g for 3 h at 4˚C, after which the supernatant was discarded and the lentiviral particles were resuspended in Roswell Park Memorial Institute 1640 (RPMI 1640; Gibco, Life Technologies, United States) culture medium. Ultimately, the obtained lentiviral particles were stored at -80 °C to be used in further experiments.

The produced lentiviruses were also titrated as previously described [6, 42]. Briefly, 1 × 10^5^ 293LTV cells were cultured in 24-well culture plates and then transduced with different dilutions of the lentiviruses using polybrene as the transduction reagent (8 µg/mL of culture medium; Sigma-Aldrich, Merck KGaA, Germany). 48 h following transduction, 293LTV cells were trypsinized, harvested, and used for DNA purification using a commercial kit (High Pure PCR Template Preparation; Sigma-Aldrich, Merck KGaA, Germany) as per the manufacturer’s protocols. Quantitative real-time PCR (qPCR) was used to assess the integrated transgene copy numbers in the purified genome of the transduced 293LTV cells, using the puromycin DNA fragment-specific forward and reverse primers as previously detailed [6, 42].

#### CAR-T cell manufacturing

Upon obtaining informed consent, human peripheral blood was collected from healthy donors (n = 3). Next, phosphate-buffered saline (PBS) was utilized to dilute the obtained blood samples with a 1:1 ratio (v/v), following which the peripheral blood mononuclear cells (PMBCs) were isolated using Ficoll-Hypaque (Lymphodex, Inno-Train, Germany) according to the manufacturer’s instructions. Primary T lymphocytes were isolated using a Pan T-Cell Isolation Kit (Miltenyi Biotec, BIOTEC GmbH, Germany) as per the manufacturer’s protocols. Dynabeads™ Human T-Activator CD3/CD28 (Gibco, Thermo Fisher Scientific, United States) were used for the activation of the primary human T cells (bead:cell ratio of 2:1), and the cells were culture and incubated for three days in complete RPMI media supplemented with interleukin 2 (IL-2; 50 IU/mL; supplemented every other day; MACS, Miltenyi Biotec, BIOTEC GmbH, Germany) in a humidified incubator with 5% CO_2_ at 37 °C. All of the experiments were in accordance with the guidelines and regulations of the *Research Ethics Committees of Tarbiat Modares University* as approved by the mentioned committees (approval ID: *IR.MODARES.REC.1400.056*).

For CAR-T cell generation, T cell transduction was carried out using the desired lentiviral particles at the multiplicity of infection (MOI) of 5 and RetroNectin^®^ (cat. No. #T100A/B; TaKaRa Bio Inc., Japan) as per the manufacturer’s protocols and detailed in our previous publications [6, 42].

#### CAR expression and surface density assessment

CAR expression rate and CAR surface density of each CAR-T cell product were assessed via flow cytometry. Briefly, FITC-labeled human CD19 (cat No. CD9-HF2H2; ACROBiosystems, Newark, DE, United States) was used for assessing both the CAR expression rate and CAR surface density of the developed CAR-T cells products based on mean fluorescence intensity (MFI). For this goal, 1 × 10^6^ of each of the transduced T cells were harvested and washed three times with ice-cold PBS. Next, the cells were incubated with FITC-labeled human CD19 as per the manufacturer’s instructions in the dark. After removing the excess CD19 molecules by washing the cells twice with PBS, the cells were resuspended in PBS (100 µL) and then stained with APC-labeled anti-human CD3 mAbs (eBioscience, San Diego, CA, United States) as per the manufacturer’s instructions to assess the percentage of T cells in each CAR-T cell batch. To remove any excess mAb, the cells were washed twice with PBS and then resuspended in PBS (200 µL) for further analysis. The cells were ultimately analyzed using a flow cytometer (BD FACSCanto II; BD Biosciences, United States), and the corresponding data were analyzed utilizing the FlowJo software (TreeStar Inc.).

#### Cytotoxicity assessment

The cytotoxic effects mediated by H85CAR-T cells, HuH85CAR-T cells, and T_mock_ cells against target cells were investigated by assessing the level of lactate dehydrogenase (LDH) as previously reported by Kang and colleagues [43]. Of note, the CD19+ cell lines Namalwa and Ramos and the CD19- cell line K562 (all obtained from the *Iranian Center for Biological Resources*, Tehran, Iran) were used as target cells for co-cultivation with H85CAR-T cells, HuH85CAR-T cell, or T_mock_ cells (transduced with the GFP lentiviral particles). Moreover, Ramos, Namalwa, and K562 cells cultured in RPMI 1640 supplemented with FBS (10%; v/v) and penicillin/streptomycin (100 I.U./mL and 100 µg/mL, respectively) as they were incubated at 37 ºC with 5% CO_2_ before being used in the experiments. Briefly, Ramos, Namalwa, and K562 cells (1 × 10^4^ cells) were seeded in 96-well tissue culture plates supplemented with 200 µL FBS-free RPMI 1640 medium and subsequently co-cultured separately with each of the effector cells at the effector to target (E:T) ratios of 1:1, 2.5:1, 5:1, and 10:1 without IL-2 supplementation for 24 h. The level of LDH was assessed in the culture media using a commercially available cytotoxicity kit (Promega; WI, United States) as per the manufacturer’s instructions. Ultimately, the absorbance value of each well was recorded using an ELISA reader instrument (Stat Fax 2100, Awareness Technology, Inc., United States) at 490 nm. The following formula was used for the calculation of specific target cell lysis percentage:

$$\eqalign{& {\rm{Target}}\,{\rm{cell}}\,{\rm{lysis}}\,\left( {\rm{\% }} \right){\rm{ = }} \cr & {{\left( \matrix{{\rm{experimental}}\,{\rm{value}}\,{\rm{ - }} \hfill \cr \,{\rm{low}}\,{\rm{control}}\,{\rm{of}}\,{\rm{effector}}\,{\rm{cells - }} \hfill \cr {\rm{low}}\,{\rm{control}}\,{\rm{of}}\,{\rm{target}}\,{\rm{cells}} \hfill \cr} \right)} \over {\left( \matrix{{\rm{high}}\,{\rm{control}}\,{\rm{of}}\,{\rm{target}}\,{\rm{cells}}\,{\rm{ - }} \hfill \cr {\rm{low}}\,{\rm{control}}\,{\rm{of}}\,{\rm{taregt}}\,{\rm{cells}} \hfill \cr} \right)}}{\rm{ \times 100}} \cr}$$

In this formula, the “*low control*” consisted of the assay medium in addition to the indicated cells whereas the “*high control*” consisted of the assay medium in addition to the indicated cells and Triton X-100 (%10).

#### Cytokine secretion assessment

**Fig. 1:**
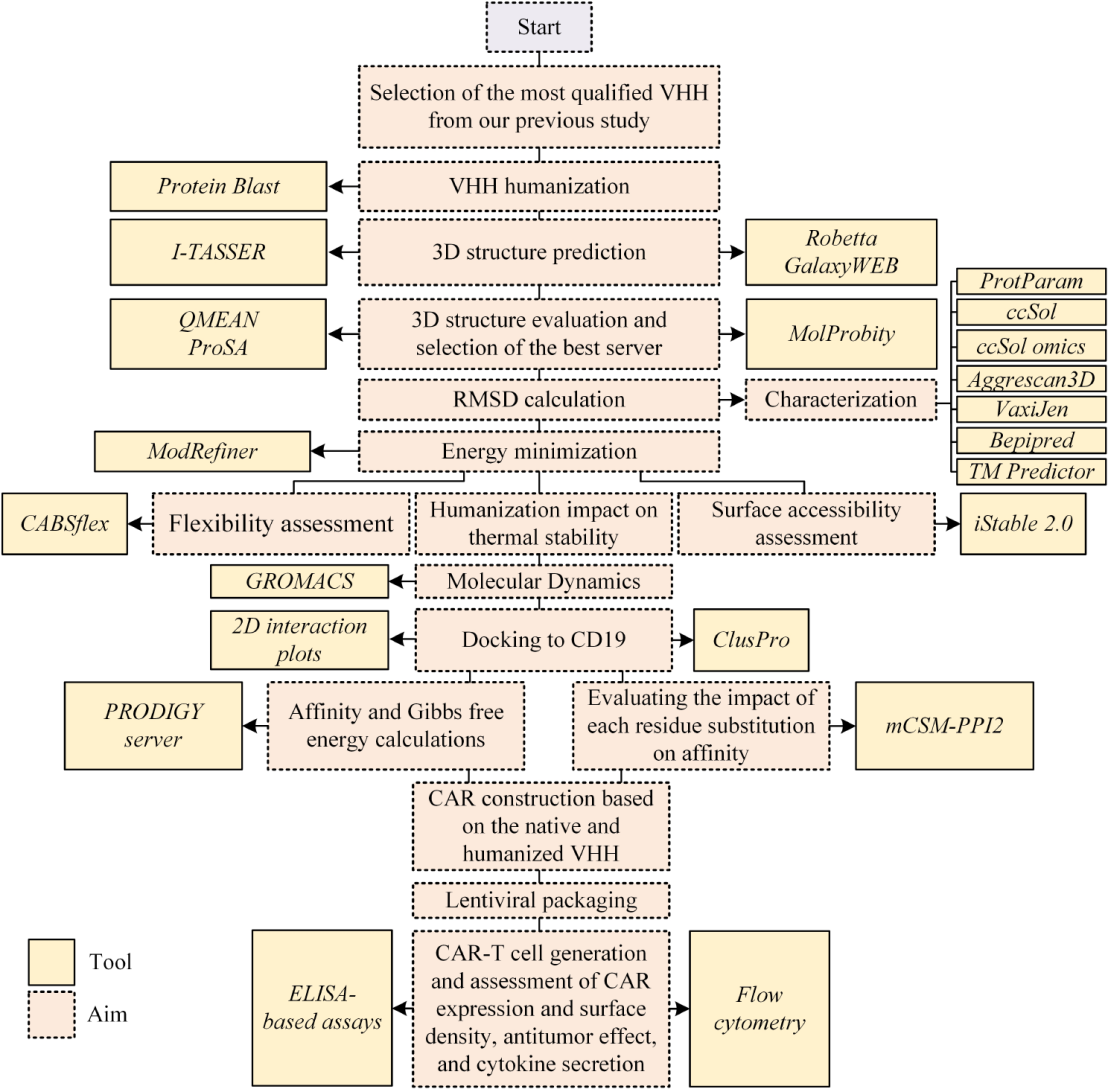
A flow chart of the in silico and in vitro experiments of this study

The level of secreted IFN-γ, IL-2, and TNF-α by H85CAR-T cells, HuH85CAR-T cell, or T_mock_ cells following their 24-h co-cultivation with Ramos, Namalwa, and K562 cells at the E:T ratio of 1:1 was assessed to further investigate the effect of the antigen-recognition domain humanization. Briefly, the supernatant from the cytotoxicity assay was collected, centrifuged at 6000 × g for 15 min to remove cells and cell debris, and ultimately used for determining the levels of IFN-γ, IL-2, and TNF-α using ELISA-based experimental kits (cat. ab236895, ab270883, and ab181421; respectively; Abcam, MA, United States) as per the manufacturer’s instructions. The flowchart of the methods and tools used in the in silico and in vitro experiments of this study is presented in Fig. 1.

### Statistical analysis

One-way and two-way ANOVA with Tukey’s multiple comparisons test and unpaired T-test were used for statistical comparison between the experimental groups. A *p-value* < 0.05 was considered statistically significant. GraphPad Prism (version 10.1.316; GraphPad Software, San Diego, CA, United States) was utilized for statistical analyses and plot illustration.

## Results

### In silico experiments

#### Humanization

**Fig. 2:**
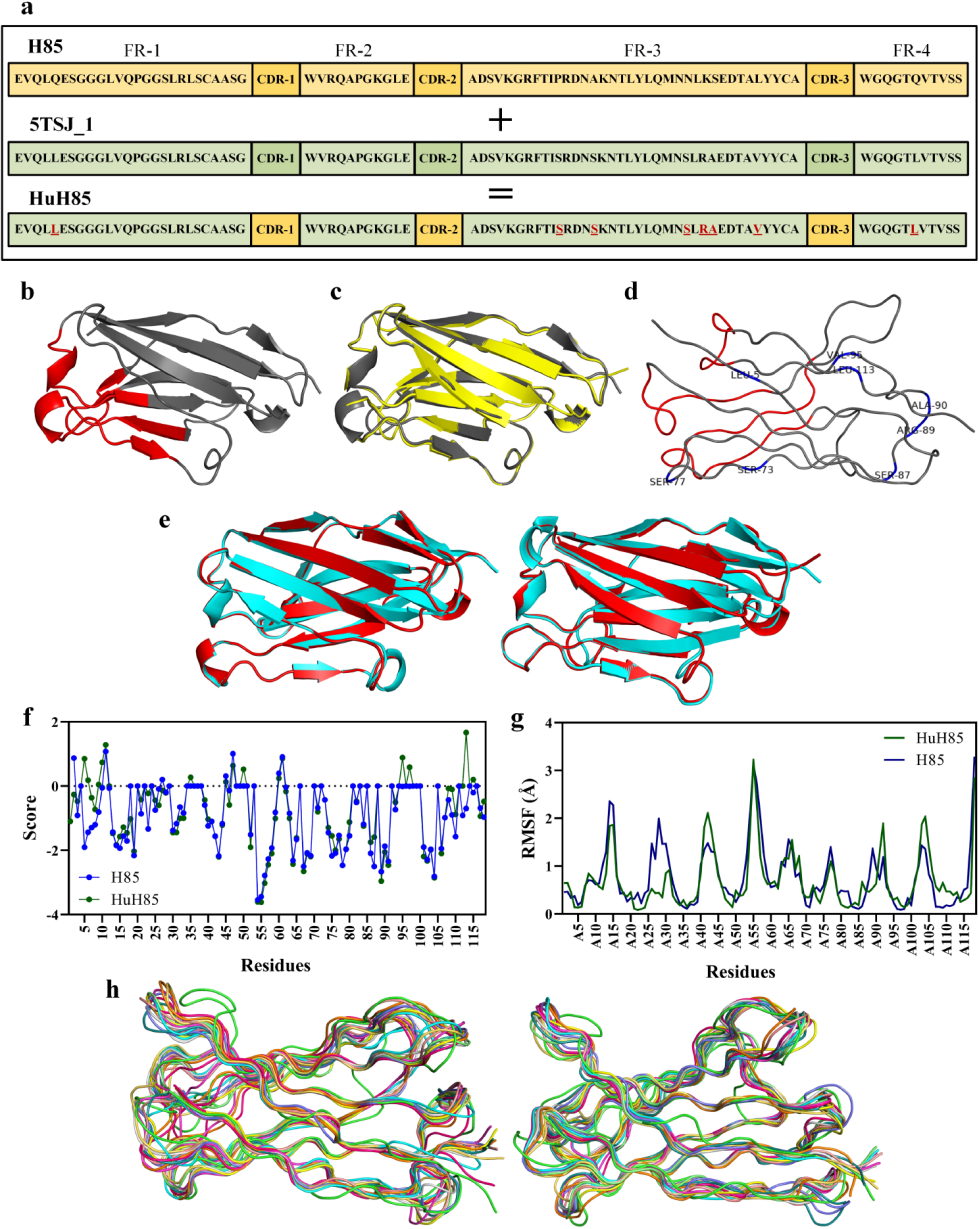
Humanization process, structure characterization, energy minimization, and flexibility simulation. **a**: Humanization of H85. All residues substituted following humanization are underlined and colored red. Amino acids are presented in one-letter codes. **b**: The best predicted 3D structure of H85 as modeled by the Robetta server. Frameworks are presented in grey whereas CDRs are in red. **c**: The confirmatory results of the alignment of H85 with HuH85 (0.313 Å) indicate that the humanization process does not negatively impact the native structure of the VHH. The native VHH is presented in grey whilst the humanized counterpart is shown in yellow. **d**: The ribbon structure of HuH85 with the substituted framework amino acids indicated by three-letter codes alongside their numbers. Intact framework residues are presented in grey whereas CDRs are in red. **e**: Cartoon presentation of the superimposed structures of H85 (left) and HuH85 (right) before (presented in red) and after (presented in cyan) the process of energy minimization. **f**: The Aggrescan3D solubility profiles of H85 and HuH85 show the solubility propensity of their corresponding 3D structures. **g**: The RMSF plot of H85 and HuH85 shows the flexibility of each VHH per residue. **h**: Protein flexibility simulation of H85 (left) and HuH85 (right). Ten 3D structures generated during the flexibility simulation process alongside the input model are superimposed as each model is presented with a different color. CDR, complementarity-determining region; FR, framework

The PDB ID of 5TSJ was selected out of a vast repertoire of human V_H_ chains because it exhibited the highest identity score to H85 (~ 76%). During the humanization process, H85 underwent eight amino acid substitutions with one in framework 1, six in framework 3, and one in framework 4 (Fig. 2a).

#### 3D structure prediction

The best predicted 3D models of each of the used servers were considered for further evaluation of structural validity. Supplementary Table 1 presents the data of the QMEANDisCo score for each predicted model (the QMEAN score has a direct relationship with the reliability of the predicted structure), the Z-Score obtained from ProSA, and the results of the Ramachandran plot evaluations of each model alongside various other profound analyses. As the results of the structural evaluation step demonstrated, the Robetta server was known to provide the most qualified predicted 3D models, which were used for the further steps of this study (Fig. 2b). An RMSD of 0.313 Å was calculated between the predicted 3D structure of H85 as aligned with HuH85 indicating that the humanization process does not impinge on the conformational structure of the VHH (Fig. 2c). Figure 2d shows the exact position of each substituted residue in the ribbon structure of HuH85.

#### Energy minimization

The results of the energy minimization process exhibited very slight changes to the 3D structures of H85 and HuH85 (Fig. 2e). An RMSD of 0.251 Å was calculated between the aligned structures of H85 before and after energy minimization whereas an RMSD of 0.335 Å was calculated between the energy-minimized structure of HuH85 as aligned with its native counterpart (with a TM-score to the initial model of 0.9961 and 0.9933 for H85 and HuH85, respectively). These results demonstrated minimal improvements in the structural conformations of H85 and HuH85.

#### Characterization

The chemical formula of H85 and HuH85 were determined alongside various other characteristics including their molecular weights, theoretical pI, half-life, instability index, aliphatic index, and grand average of hydropathicity, all of which were computed by the ProtParam server (Table 1). These results demonstrated that the humanization process does not negatively impact the VHH in terms of its physicochemical properties. Furthermore, since camelid antibodies contain hydrophilic amino acids in their framework regions, it is reasonable to expect that the substitution of such amino acids with their human counterparts would result in the partial or complete loss of antibody solubility. The results of the ccSol server were in compliance with the above-mentioned expectation as a slight decrease in the solubility probability of H85 was predicted following its humanization (Table 2). The results of the ccSol omics server also predicted a similar pattern in the solubility probability of H85 following its humanization as expected (Table 2). The results of the Aggrecan3D server were also consistent with the predictions of the ccSol and ccSol omics servers, as an increase in the average solubility and total solubility scores of H85 were predicted following its humanization (Table 2; Fig. 2f); however, it is safe to assert that the humanization process does not impact the aggregation propensity of H85 in an erratic fashion according to both of these values.

**Table 1:** Characteristics and properties of H85 and HuH85 estimated or calculated by ProtParam

VHH	Molecular weight	Theoretical pI	Chemical formula	Estimated half-life (hours)			Instability index (stability state)	Aliphatic index	Grand average of hydropathicity (GRAVY)
				In vitro	In yeast	In *E. coli*			
H85	12874.27	5.02	C_565_H_868_N_154_O_183_S_4_	1	0.5	> 10	39.05 (stable)	66.10	-0.450
HuH85	12821.25	5.02	C_563_H_869_N_153_O_182_S_4_	1	0.5	> 10	38.53 (stable)	71.86	-0.298

**Table 2:** The details of the solubility profile and aggregation propensity of H85 and HuH85. In the Aggrescan3D server results, more negative values indicate higher normalized solubility (threshold set at 0)

Test		H85	HuH85
ccSol (% of solubility probability)		22	13
ccSol omics (% of solubility probability)		67	25
Aggrescan3D	Minimal score value	-3.5416	-3.6106
	Maximal score value	1.0797	1.6699
	Average score	-0.882	-0.7677
	Total score value	-103.1968	-90.5921

According to the results of VaxiJen, the overall antigenicity index of H85 experienced a substantial decrease after humanization indicating the success of the humanization process in reducing the antigenicity of the VHH (Table 3). Despite still being designated as an “*antigen”* by the server after humanization, H85 exhibited a substantial decrease in its antigenicity index which is close to being below the defined threshold of “*antigen*”. The immunological analyses of the Bepipred linear epitope prediction tool indicated that H85 and HuH85 are free of any potential B-cell epitopes; however, a slight increase in the average value attributed to HuH85 was predicted (Table 3). Moreover, the predictions of the Tm Predictor server indicated no substantial change in the melting temperature of the VHH as a result of humanization as a Tm of 55 ~ 65 ºC was predicted for both H85 and HuH85.

**Table 3:** The antigenicity index of H85 and HuH85 as predicted by VaxiJen and the results of the Bepipred linear epitope prediction tool. The average scores of H85 and HuH85 were estimated with the threshold set at 0.500

Test		H85	HuH85
VaxiJen server	Prediction value	0.5585	0.5064
	Antigenicity state	Antigen	Antigen
Bepipred Linear Epitope Prediction	Average	0.474	0.478
	Minimum	0.215	0.214
	Maximum	0.660	0.655

#### Flexibility, thermal stability, and surface accessibility assessments

According to the results of the CABSflex server, both H85 and HuH85 exhibited a similar pattern of flexibility as no sharp spikes of root-mean-square fluctuation (RMSF) were observed in the structure of HuH85 as a result of framework amino acid substitution in the humanization process (Fig. 2g and h). Analysis of the RMSF plot indicated that it is safe to conclude that these amino acid substitutions do not majorly impact the structural conformation of the humanized VHH.

Moreover, the exact contribution of each framework residue substitution to the thermal stability of HuH85 was predicted by iStable 2.0 (at 37.0 ℃ and the pH of 7.40, which is the normal pH of the blood). The results predicted that half of the residue substitutions would increase the thermal stability of the humanized VHH while the other half would contribute to its decrease (Table 4). Furthermore, analysis of the surface accessibility and relative surface accessibility plots for H85 and HuH85 indicated no major change in the surface accessibility profiles of the VHH as a result of humanization (data not shown).

**Table 4:** A detailed contribution of each framework amino acid substitution to the increasing or decreasing of the thermal stability of HuH85 (predicted at 37.0 ℃ and the pH of 7.40)

Mutation	Thermal stability
Q5L	Increase
P73S	Decrease
A77S	Decrease
N87S	Decrease
K89R	Increase
S90A	Increase
L95V	Decrease
Q113L	Increase

#### MD analysis

**Fig. 3:**
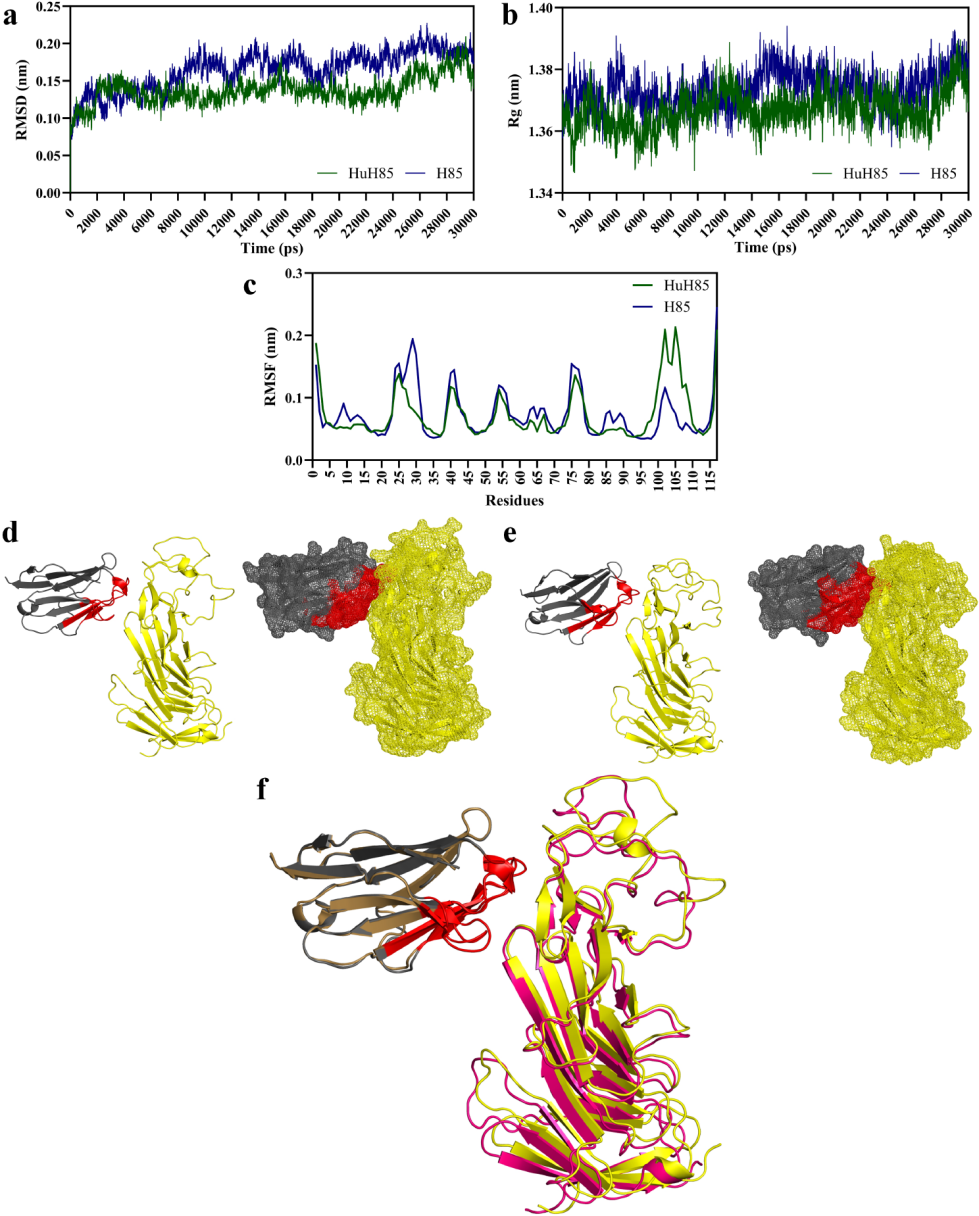
Molecular dynamics (MD) and the docking complexes of H85 and HuH85 to CD19. **a**: The RMSD values of H85 and HuH85 plotted as a function of time over the course of the MD simulation. **b**: The R_g_ values of H85 and HuH85 plotted as a function of time over the course of the MD simulation. **c**: The RMSF plot of H85 and HuH85 shows the flexibility of each VHH per residue over the course of the MD simulation. Of note, each nm equals 10 Å. **d** and **e**: The ClusPro server docking complexes of H85 and HuH85 as docked to CD19, respectively. The complex on the left is in cartoon presentation while the same complex is presented in mesh presentation on the right. Frameworks are presented in grey, CDRs in red, and CD19 in yellow. **f**: The aligned structure of the docking complex of H85 (frameworks in grey and CDRs in red) to CD19 (yellow) to the docking complex of HuH85 (frameworks in sand and CDRs in red) to CD19 (pink)

The RMSD values of H85 and HuH85 were plotted as a function of time to simply show the deviation of each VHH structure at different simulation times (Fig. 3a). The results indicate that the backbones of both of our native and humanized VHHs are stable and they do not exhibit considerable spikes of high RMSD values over the course of the MD simulation. Moreover, HuH85 exhibited lower RMSD values over the course of the simulation runs indicating that the humanized format of the VHH is more stable in comparison with its native counterpart. Additionally, further stability analysis was also carried out using the R_g_ plot of H85 and HuH85 as a function of simulation time (Fig. 3b). The R_g_ is an indicator of the compactness of any given protein structure in a way that larger R_g_ values are indicators of less tightly packed protein structures and that any given stably-folded protein will tend to maintain a relatively steady value of R_g_ over the course of the MD simulation. According to the R_g_ values of H85 and HuH85, both VHHs very well manage to maintain their folded structure (compactness) over the course of 30 ns. In a comparative view, HuH85 exhibited relatively steadier R_g_ values over the course of the simulation in comparison with H85. In reference to the flexibility of H85 and HuH85 in the MD simulation step, the results were consistent with those of the CABSflex server. Briefly, H85 and HuH85 structures exhibited similar flexibility patterns with no sharp fluctuations that could be constructed as a negative impact as a result of humanization (Fig. 3c).

#### CD19-binding efficacy assessments

**Fig. 4:**
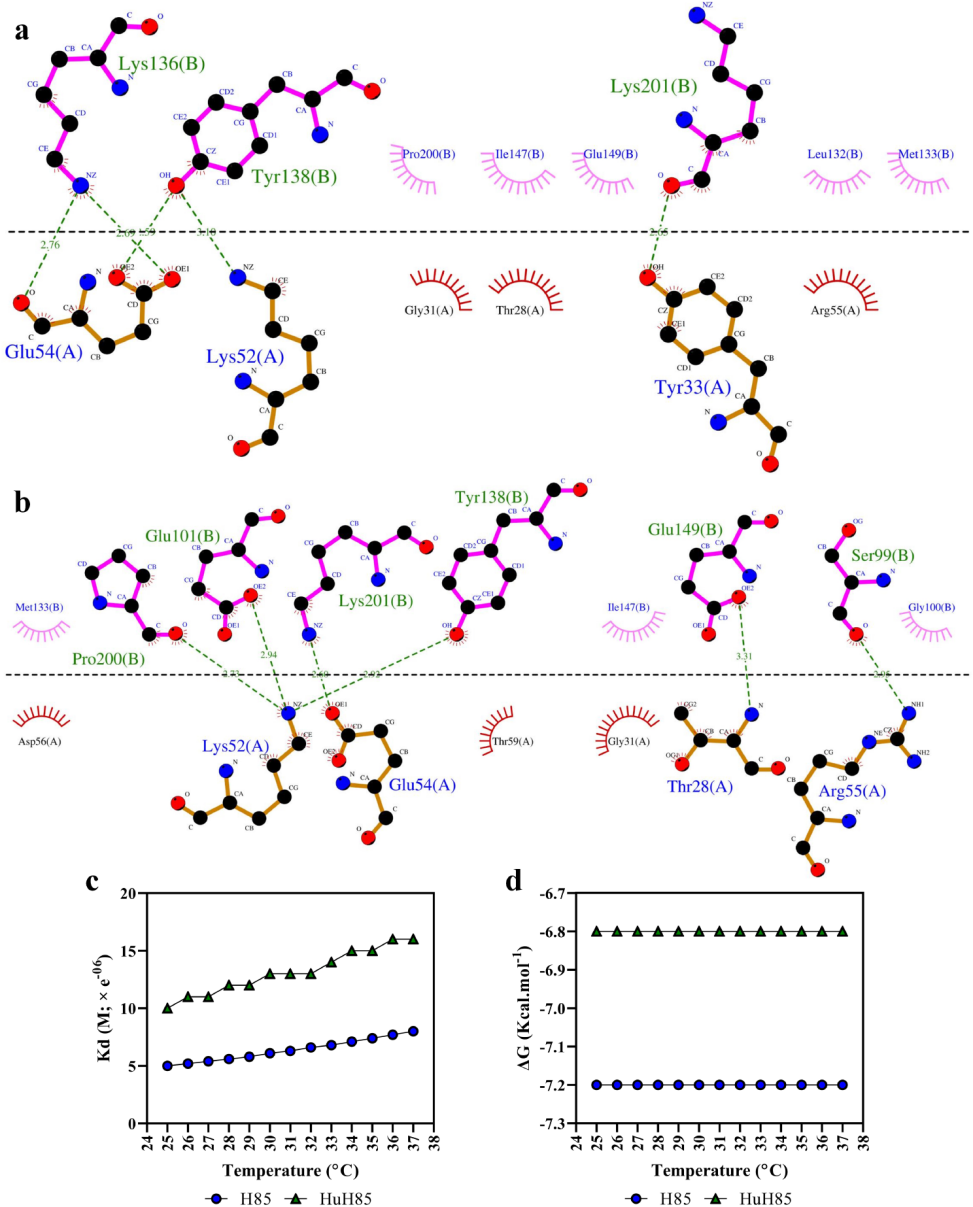
2D interaction plots and the predicted effects of temperature increase on the predicted K_d_ and ΔG. **a** and **b**: 2D interaction plots of the binding of H85 and HuH85 to CD19, respectively. The residues placed above the horizontal dashed line (antigen-antibody interface) represent those of CD19 (B chain) whereas those below the line represent the residues of the VHH (A chain). Arcs and dashed lines represent hydrophobic contacts and hydrogen bonds, respectively. **c** and **d**: The impact of increasing temperature from 25 to 37 ºC on the K_d_ and ΔG of H85 and HuH85 as docked to CD19, respectively

The ClusPro server was selected to carry out the docking step since this server utilizes a hybrid algorithm of template-based homology modeling and ab initio-free docking for predicting the binding of certain receptors to ligands. The docking results confirmed the previous steps of our experiment by indicating that the humanization of H85 does not impinge on its ability to bind CD19 (Fig. 3d, e). Moreover, these findings indicated that HuH85 targets CD19 in a similar orientation, and probably at the same epitope, as compared to H85 (Fig. 3f). To further identify the interactive residues of H85 and HuH85 that participate in binding CD19, their corresponding 2D interaction plots were illustrated (Fig. 4a, b, respectively). According to the results, HuH85 targets a similar epitope of CD19 as compared with H85 with identical CDR residues. Briefly, Tyr138, Pro200, Ile147, Glu149, Lys201, and Met133 were residues of CD19 participating in the docking of both H85 and HuH85 to CD19. Moreover, Glu54, Lys52, Gly31, Thr28, and Arg55 were mutual VHH CDR residues that participated in the binding of both H85 and HuH85 to CD19.

Moreover, besides the goal of reducing the immunogenicity of animal-origin mAbs, any successful humanization process is one that does not result in the loss of antigen-affinity of the antibody following humanization. Since antibody affinity and K_d_ are inversely related, any high-affinity antibody-antigen interaction is characterized by a low K_d_ value. As the results of the PRODIGY server indicated both H85 and HuH85 experienced a slight K_d_ increase following a temperature increase from 25 to 37 ºC (Fig. 4c). In the case of H85, the K_d_ elevated from 5e^− 06^ to 8e^− 06^ M whereas an increase from 10e^− 06^ to 16e^− 06^ M was predicted for HuH85 as the temperature increased from 25 to 37 ºC. Additionally, the results of the PRODIGY server further validated the docking reactions by predicting their Gibbs free energy indicating that H85 and HuH85 bind CD19 in an energetically favorable state (Fig. 4d). Moreover, no fluctuations were predicted for the docking complexes of H85 to CD19 and HuH85 to CD19 as temperature increased from 25 to 37 ºC.

Moreover, the mCSM-PPI2 server predicted the exact contribution of each framework amino acid substitution to the increasing or decreasing of HuH85 affinity to CD19 (Table 5). According to the results, five residue substitutions were predicted to increase the affinity of HuH85 to CD19 whereas the three remaining residue substitutions were predicted to decrease affinity.

**Table 5:** The increasing or decreasing impact of each amino acid substitution on the affinity of HuH85 to CD19

Wild Type	Residue number	Mutant	Predicted ΔΔG^Affinity^ (kcal/mol)	Affinity
GLN	5	LEU	-0.013	Decreasing
PRO	73	SER	0.149	Increasing
ALA	77	SER	0.099	Increasing
ASN	87	SER	-0.028	Decreasing
LYS	89	ARG	0.060	Increasing
SER	90	ALA	-0.031	Decreasing
LEU	95	VAL	0.115	Increasing
GLN	113	LEU	0.094	Increasing

### In vitro experiments

#### CAR construction and lentiviral packaging

**Fig. 5:**
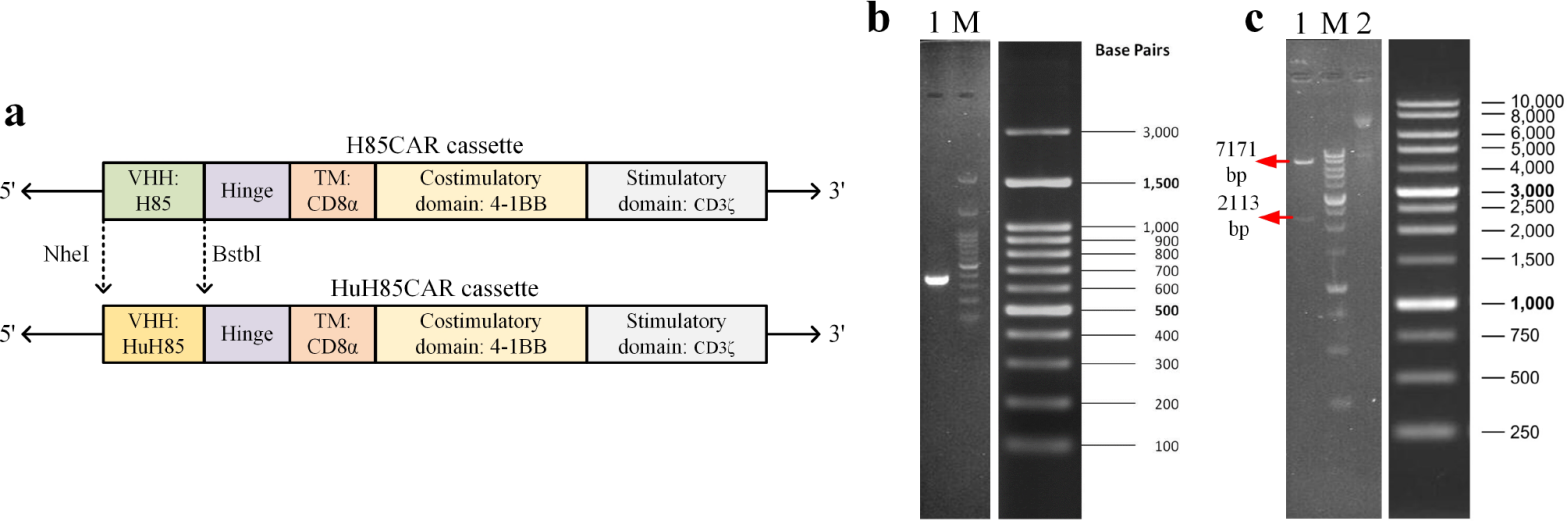
CAR cassettes and restriction cloning of the HuH85-encoding fragment. **a**: A simplified schematic of the H85CAR construct which is modified for the insertion of the HuH85-encoding DNA fragment only to develop the HuH8CAR construct. **b**: PCR-amplification of the HuH85-encoding fragment electrophoresed on 1% agarose gel. Lane 1: PCR amplicon (380 bp); Lane M: 100 bp DNA marker. **c**: Enzymatic digestion of the HuH85CAR-pLJM1 vector electrophoresed on 1% agarose gel for the verification of the HuH8CAR cassette construction using KpnI. Lane 1: KpnI-digested HuH85CAR-pLJM1 vector; Lane M: 1000 bp DNA maker; Lane 2: undigested HuH85CAR-pLJM1 vector

The HuH85CAR construct with HuH85 as its antigen-recognition domain was successfully developed. Figure 5a presents a simplified schematic illustration of H85CAR and HuH85CAR. Briefly, the HuH85-encoding DNA fragment was successfully PCR-amplified using the previously mentioned primers and verified on 1% agarose gel (a 380 bp band) (Fig. 5b). Moreover, the replacement of the HuH85-encoding DNA fragment with that of H85 was verified through DNA sequencing (data not included) and enzymatic digestion of the pLJM1-HuH85CAR vector with KpnI as 7171 and 2113 bp DNA fragment bands were observed 1% agarose gel (Fig. 5c). Furthermore, the lentiviral packaging step was verified by assessing the expression of GFP by 293LTV cells transfected with the PLJM1-EGFP transfer plasmid under a fluorescence microscope at different indicated time points (data not included).

#### CAR expression and surface density assessment

**Fig. 6:**
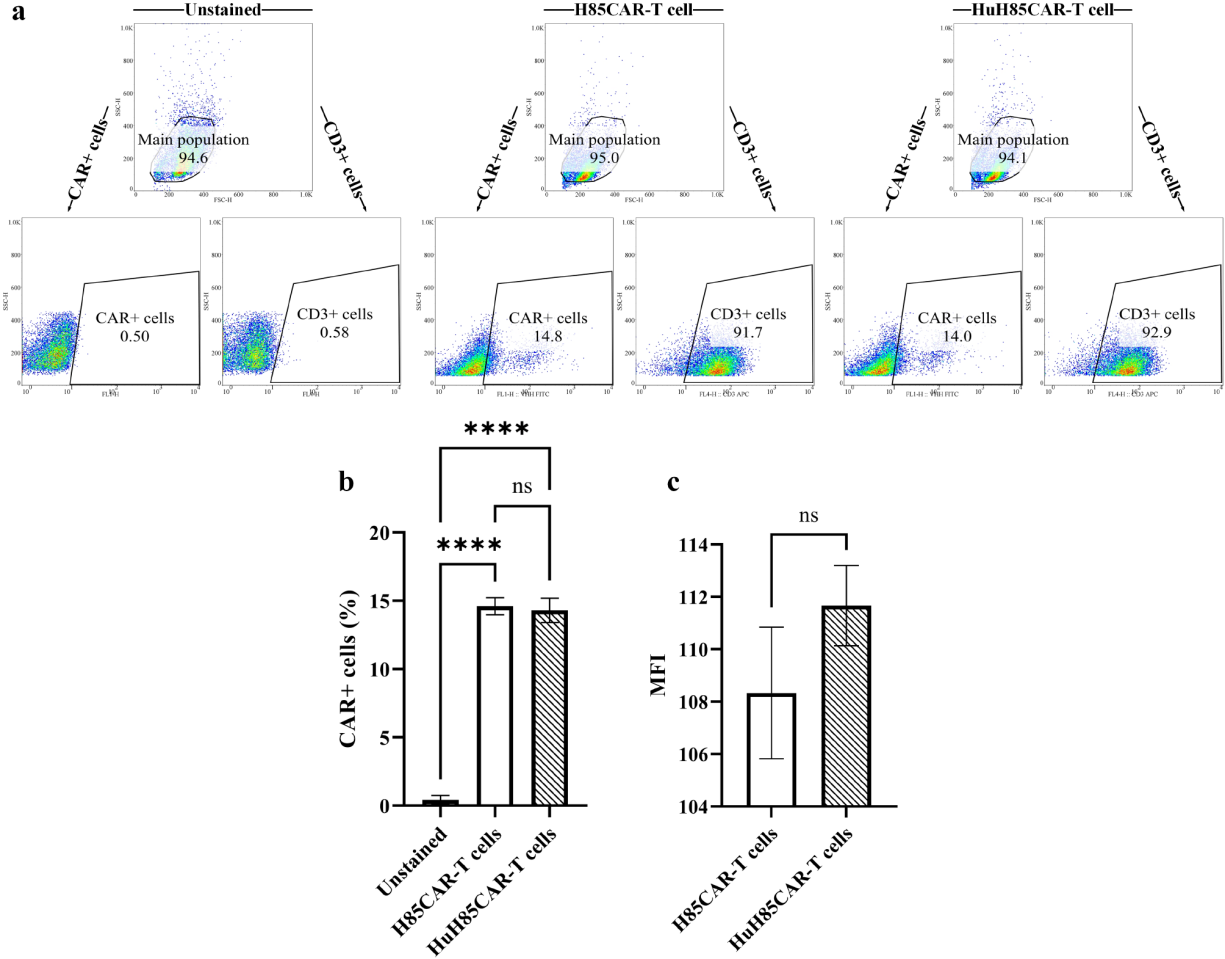
Flow cytometric analysis of H85CAR-T cells and HuH85CAR-T cells. **a**: Flow cytometry scatter plots of H85CAR-T cells and HuH85CAR-T cells. The cells were gated on the main population and then the rates of CD3+ cells or CAR+ cells were assessed within that gate. **b**: Statistical comparison in the percentage of CAR+ cells in the H85CAR-T cell, HuH85CAR-T cell, and the unstained groups. **c**: Statistical comparison of the MFI of the FL1-H channel in the H85CAR-T cell and HuH85CAR-T cell groups. MFI, mean fluorescence intensity. **** and ns represent *p* < 0.0001 and *p* > 0.05, respectively. Data are expressed as the mean ± standard deviation. All experiments have been carried out in triplicate (*n* = 3)

According to the flow cytometric assessments, ~ 92% of cells were proficient in the expression of CD3 which validates the process of T cell isolation (Fig. 6a). Moreover, there was no significant difference in the CAR expression rates in the H85CAR-T cell and HuH85CAR-T cell groups (14.6 ± 0.62 vs. 14.3 ± 0.88%, respectively; *p* > 0.05), paving the way for their MFI comparison (Fig. 6a and b). A comparison of the MFI values of the H85CAR-T cells and HuH85CAR-T cells demonstrated that these two CAR-T cell products were capable of expressing their corresponding CAR molecules at a comparable cell surface density (108.33 ± 2.51 vs. 111.66 ± 1.52, respectively; *p* > 0.05), meaning that the VHH humanization did not negatively impact CAR expression (Fig. 6c).

#### Cytotoxicity assessment

**Fig. 7:**
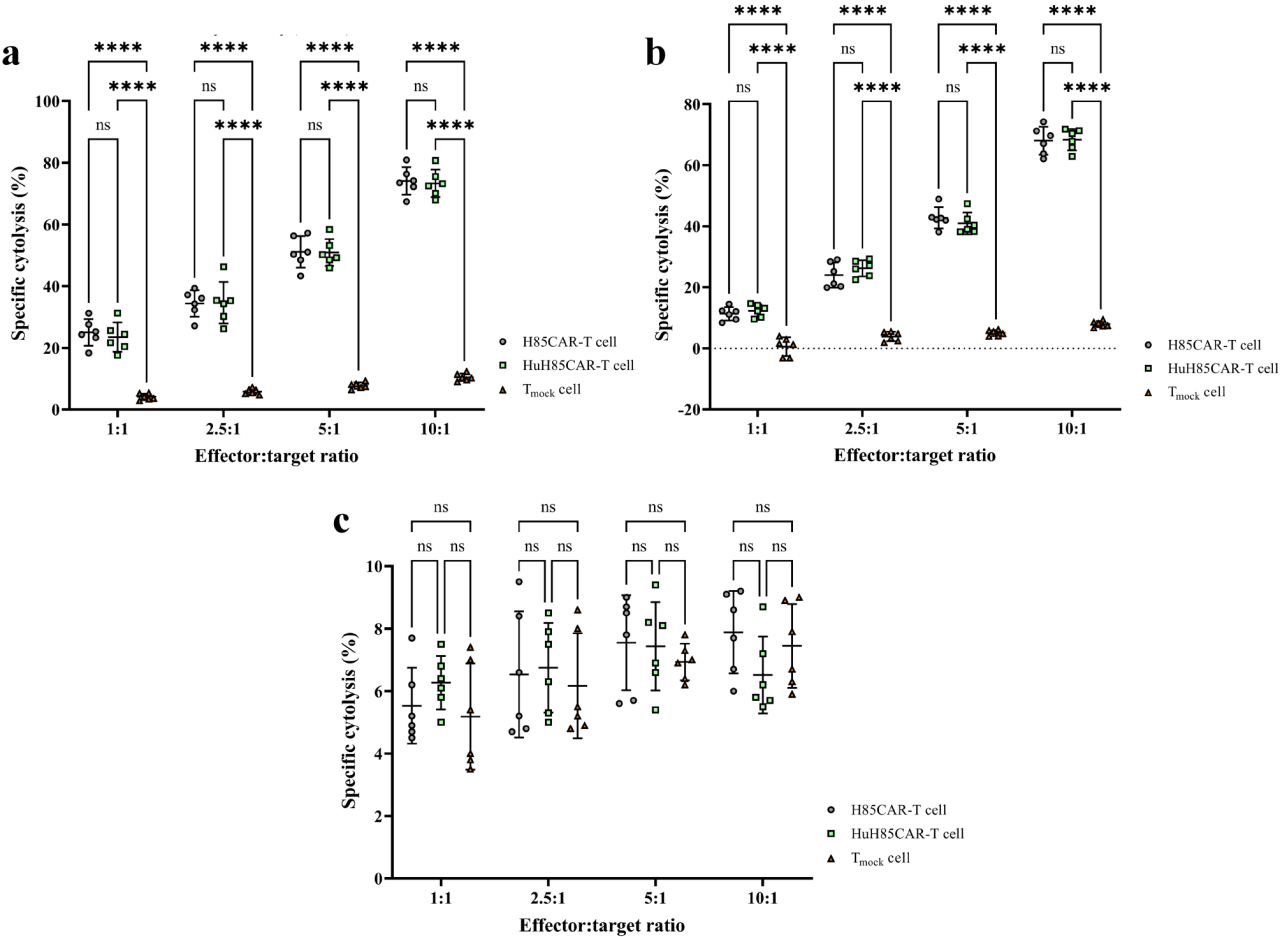
Assessment of the cytotoxic effects of different effector cells mediated against Ramos (**a**), Namalwa (**b**), and K562 (**c**) cells at four different effector:target cell ratios following their 24-h co-cultivation. **** and ns represent *p* < 0.0001 and *p* > 0.05, respectively. Data are expressed as the mean ± standard deviation. All experiments have been carried out in triplicate (*n* = 6)

The results of the cytotoxicity assay demonstrated that H85CAR-T cells and HuH85CAR-T cells mediated comparable levels of cytotoxicity against the target cells (Fig. 7). In the Ramos co-culture group (Fig. 7a), HuH85CAR-T cells mediated comparable rates of cytotoxicity against the target cells following a 24-h co-cultivation as compared with H85CAR-T cells at the E:T ratios of 1:1, 2.5:1, 5:1, and 10:1 (23.50 ± 4.76 vs. 25.01 ± 4.32, 34.62 ± 6.75 vs. 34.39 ± 4.28, 50.90 ± 4.37 vs. 51.10 ± 5.14, and 73.32 ± 4.46 vs. 74.08 ± 4.47%, respectively; *p* > 0.05 for all comparisons). Moreover, a similar pattern but with lower rates of cytotoxicity against the target cells was observed in the Namalwa co-culture group as HuH85CAR-T cells exerted similar cytolytic reactions in comparison with H85CAR-T cells at the E:T ratios of 1:1, 2.5:1, 5:1, and 10:1 (12.32 ± 2.06 vs. 11.32 ± 2.17, 26.20 ± 2.65 vs. 24.02 ± 4.12, 40.93 ± 3.53 vs. 42.78 ± 3.47, and 68.33 ± 3.50 vs. 68.02 ± 4.57%, respectively; *p* > 0.05 for all comparisons) (Fig. 7b). In the Ramos co-culture group, both HuH85CAR-T cells (23.50 ± 4.76 vs. 4.13 ± 1.03, 34.62 ± 6.75 vs. 5.85 ± 0.93, 50.90 ± 4.37 vs. 7.76 ± 1.01, and 73.32 ± 4.46 vs. 10.47 ± 1.20%, respectively; *p* < 0.0001 for all comparisons) and H85CAR-T cells (25.01 ± 4.32 vs. 4.133 ± 1.03, 34.39 ± 4.28 vs. 5.85 ± 0.93, 51.10 ± 5.14 vs. 7.76 ± 1.01, and 74.08 ± 4.47 vs. 10.47 ± 1.20%, respectively; *p* < 0.0001 for all comparisons) mediated significantly higher rates of target cell lysis as compared with T_mock_ cells at the E:T ratios of 1:1, 2.5:1, 5:1, and 10:1. Furthermore, a similar cytotoxicity pattern was also observed against Namalwa cells as HuH85CAR-T cells (12.31 ± 2.06 vs. 0.55 ± 3.06, 26.2 ± 2.65 vs. 3.86 ± 1.54, 40.93 ± 3.53 vs. 5.03 ± 0.95, and 68.33 ± 3.50 vs. 7.95 ± 1.01%, respectively; *p* < 0.0001 for all comparisons) and H85CAR-T cells (11.31 ± 2.17 vs. 0.55 ± 3.06, 24.01 ± 4.12 vs. 3.86 ± 1.54, 42.78 ± 3.47 vs. 5.03 ± 0.95, and 68.01 ± 4.57 vs. 7.95 ± 1.01%, respectively; *p* < 0.0001 for all comparisons) in comparison with T_mock_ cells at the E:T ratios of 1:1, 2.5:1, 5:1, and 10:1 following 24 h of co-cultivation.

In the K562 co-culture group (Fig. 7c), no significant difference was observed in the rate of cytotoxicity against the target cells mediated by any of the effector cells at any of the investigated E: T ratios. Briefly, HuH85CAR-T cells mediated comparable rates of cytotoxicity against K562 cells after 24 h of co-cultivation as compared with H85CAR-T cells (6.26 ± 0.85 vs. 5.53 ± 1.21, 6.75 ± 1.43 vs. 6.53 ± 2.02, 7.43 ± 1.41 vs. 7.55 ± 1.52, and 6.51 ± 1.22 vs. 7.88 ± 1.31% at the E: T ratios of 1:1, 2.5:1, 5:1, and 10:1, respectively; *p* > 0.05 for all comparisons) and T_mock_ cells (6.26 ± 0.85 vs. 5.18 ± 1.69, 6.75 ± 1.43 vs. 6.16 ± 1.68, 7.43 ± 1.41 vs. 6.93 ± 0.58, and 6.51 ± 1.22 vs. 7.45 ± 1.34% at the E: T ratios of 1:1, 2.5:1, 5:1, and 10:1, respectively; *p* > 0.05 for all comparisons). Moreover, a similar pattern was observed in the comparisons between H85CAR-T cells vs. T_mock_ cells (5.53 ± 1.21 vs. 5.18 ± 1.69, 6.53 ± 2.02 vs. 6.16 ± 1.68, 7.55 ± 1.52 vs. 6.93 ± 0.58, and 7.88 ± 1.31 vs. 7.45 ± 1.34% at the E: T ratios of 1:1, 2.5:1, 5:1, and 10:1, respectively; *p* > 0.05 for all comparisons).

#### Cytokine secretion assessment

**Fig. 8:**
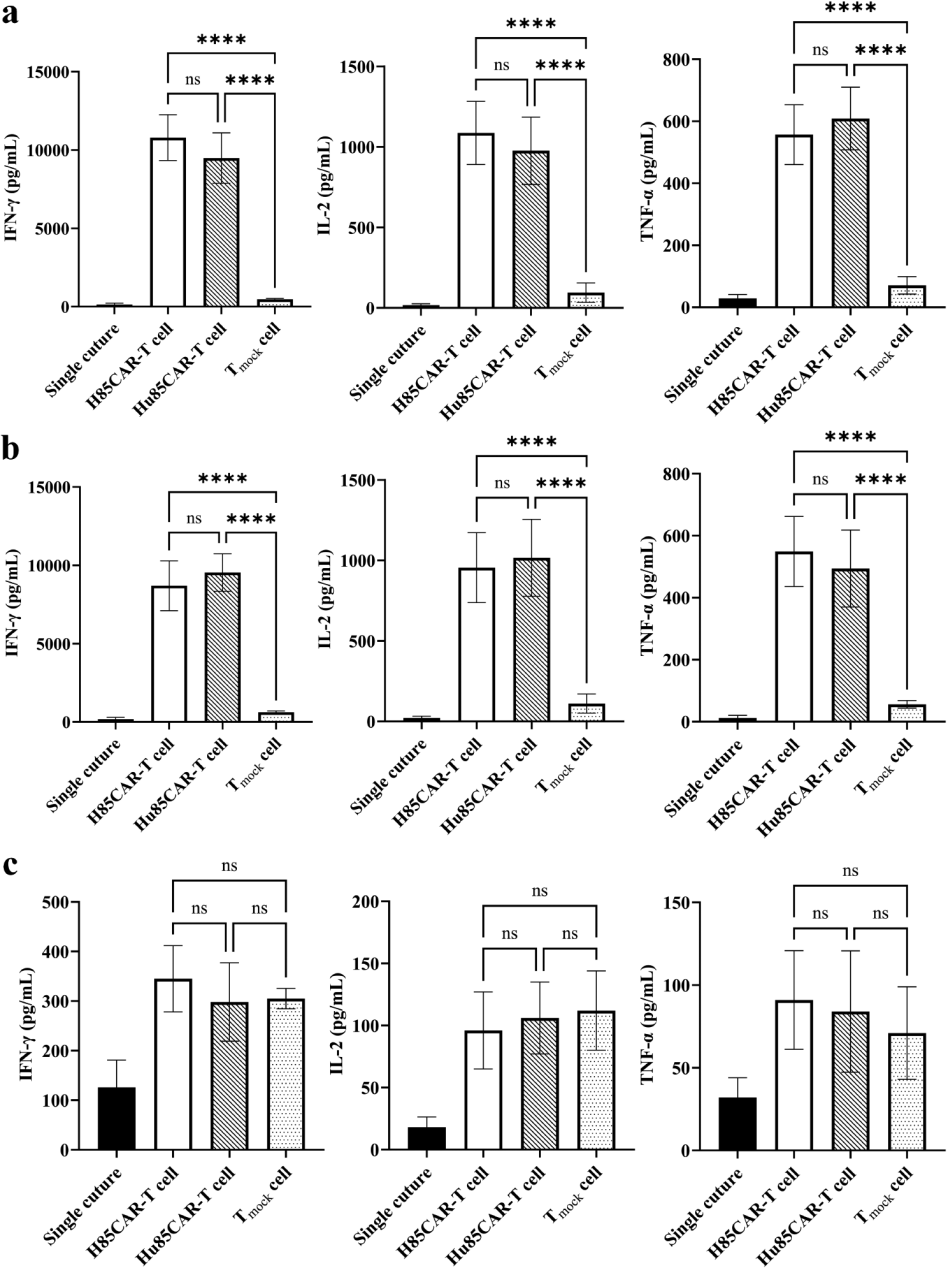
The levels of secreted IFN-γ, IL-2, and TNF-α following the co-cultivation of H85CAR-T cells, HuH85CAR-T cells, or T_mock_ cells with Ramos (**a**), Namalwa (**b**) or K562 (**c**) cells at the E:T ratio of 1:1. **** and ns represent *p* < 0.0001 and *p* > 0.05, respectively. Data are expressed as the mean ± standard deviation. All experiments have been carried out in triplicate (*n* = 6)

To further investigate the impact of antigen-recognition domain humanization on the functionality of the engineered T cells, H85CAR-T cells, HuH85CAR-T cells, and T_mock_ cells were co-cultured with Ramos, Namalwa, and K562 cells, and the levels of secreted cytokines were measured after 24 h. According to the results (Fig. 8a), HuH85CAR-T cells secreted comparable levels of IFN-γ, IL-2, and TNF-α in comparison with H85CAR-T cells in the Ramos co-cultivation group (9481 ± 1609 vs. 10,790 ± 1466, 976 ± 209 vs. 1087 ± 196, and 609 ± 101 vs. 557 ± 96.3 pg/mL, respectively; *p* > 0.05 for all comparisons). A similar pattern was observed in the Namalwa co-cultivation group as there was no significant difference in the levels of IFN-γ, IL-2, and TNF-α secreted by HuH85CAR-T cells compared with those of H85CAR-T cells (9540 ± 1200 vs. 8700 ± 1591, 1016 ± 239 vs. 955 ± 217, 494 ± 124 vs. 549 ± 113 pg/mL, respectively; *p* > 0.05 for all comparisons) (Fig. 8b). The results of the K562 co-cultivation group indicated that the levels of IFN-γ, IL-2, and TNF-α secretion of T_mock_ cells upon encountering CD19- cells are comparable to those of H85CAR-T cells (305 ± 20.6 vs. 345 ± 67, 112 ± 32 vs. 96 ± 31, and 71 ± 28 vs. 91 ± 29.8 pg/mL, respectively; *p* > 0.05 for all comparisons) and HuH85CAR-T cells (305 ± 20.6 vs. 298 ± 79, 112 ± 32 vs. 106 ± 29, and 71 ± 28 vs. 84 ± 36.7 pg/mL, respectively; *p* > 0.05 for all comparisons) (Fig. 8c).

## Discussion

CAR-T cells have undoubtedly proven a promising treatment option for particular groups of patients with R/R B-ALL, DLBCL, and MCL, and follicular lymphoma (FL) as four CD19-redirected products have been granted US FDA approval for medical use [44]. But looking at this clinical success with a magnifying glass might result in finding some flaws that if taken into consideration might increase the clinical applicability of CAR-T cells in a way that more patients can benefit from this treatment modality. Recently, numerous studies have reported the presence of neutralizing antibodies against the antigen-recognition domains of anti-CD19 or anti-CAIX CAR-T cells in the respective recipients which contributes to the abrupt clearance of the relative CAR-T cells shortly after the first round of administration [45, 46]. This rapid clearance results in the abrogation of all CAR-T cell-related tumoricidal responses and increases the risk of disease relapse, especially in patients with a high disease burden who require more than one round of CAR-T cell infusion [45–48]. Alongside using fully human antigen-recognition domains in CAR constructs, another potential strategy for counteracting such immune responses is reducing the immunogenicity of such antigen-recognition domains through their humanization [51, 52]. This strategy substantially increases the therapeutic index of CAR-T cells by enhancing the longevity of the adoptively transferred T cells and it has so far been investigated in both hematologic malignancies and solid tumors [49, 56, 57]. Aside from scFvs, VHHs have also been employed as the antigen-recognition domains of CAR-T cells owing to their ideal characteristics such as their small size, their high stability and solubility, and their ability to recognize hidden antigen epitopes, because of their CDR3 loop which is relatively longer than those of mouse or human antibodies [58]. Herein, a CD19-specific VHH previously isolated in our laboratory using the phage display technique was humanized using in-depth in silico approaches [10]. The in silico findings indicated that the humanization process does not impinge on the binding capacity of the VHH to CD19; however, to further corroborate these findings, CAR constructs based on the native and humanized VHH were developed and primary T cells were transduced for their expression. The results of the in vitro experiments validated what had been predicted in the in silico experiments as H85CAR-T cells and HuH85CAR-T cells exhibited comparable CAR expression rates and surface density as assessed via flow cytometry. Moreover, H85CAR-T cells and HuH85CAR-T mediated comparable antitumor effects alongside secreting comparable levels of IFN-γ, IL-2, and TNF-α upon co-cultivation with the CD19+ cell lines Ramos and Namalwa indicating the CD19-dependent reactivity of both CAR-T cell products. Further analysis of the rate of antitumor reactivity and the levels of the secreted cytokines demonstrated that both H85CAR-T cells and HuH85CAR-T cells exhibit a negligible degree of cross-reactivity toward a CD19- cell line, K562, comparable to those of T_mock_ cells. Our results were consistent with the findings of other studies which demonstrated that humanization of the antigen-recognition domain of CAR-T cells does not diminish their binding ability to their indicated antigen [56]. In the first pilot/phase I study that investigated the anti-leukemic efficacy of humanized CAR-T cells in children and young adults with B-ALL, Maude and colleagues reported that 100% of the patients that had not undergone any previous CAR-T cell treatment and 64% of the patients previously treated with murine CAR-T cells achieved remission [56]. According to another clinical investigation (NCT02782351), 13 out of 14 patients (92.9%) who received a single dose of humanized CD19-redirected CAR-T cells (named Hu19-CAR-Ts), after lymphodepletion with cyclophosphamide and fludarabine and without prior CAR-T cell treatment, achieved complete remission (CR) or CR with incomplete count recovery (CRi) [49]. The same study further highlighted the capability of Hu19-CAR-Ts in mediating remission in R/R ALL patients when 1 out of 3 patients who had previously failed a second infusion of murine CAR-T cells achieved CR after treatment with Hu19-CAR-Ts [49]. Another study (ChiCTR1800017401) on two newly diagnosed untreated B-ALL patients also reported that a single infusion of Hu19-CAR-Ts administered after lymphodepletion with fludarabine and cyclophosphamide culminated in the prolonged remission of the patients [54]. According to another study that developed humanized selective CD19-redirected CAR-T cells (termed hsCAR-Ts), their humanized CAR exhibited a 6-fold higher affinity to CD19, as compared with the murine counterpart [53]. Moreover, 4 out of 5 R/R B-ALL patients, who had previously relapsed after receiving murine CAR-T cells, achieved complete molecular remission after receiving hsCAR-Ts, one of whom had an extramedullary disease that had engaged the central nervous system [53]. Furthermore, no antibodies reactive to hsCAR were present in the sera of the patients, which further emphasizes that there will be no obstacles for repetitive infusions of hsCAR-Ts, if necessary [53]. CAR-T cells with humanized antigen-recognition domains have also been investigated in the context of solid tumors. According to an investigation, Johnson and colleagues developed a series of humanized scFvs specific for EGFRvIII and designed and developed 2nd -generation CAR-T cells which were able to mediate pronounced tumor outgrowth suppression in xenograft models of human glioblastoma proficient in the expression of EGFRvIII. Johnson and colleagues also reported the initiation of a clinical investigation (NCT02209376) in which individuals with EGFRvIII+ glioblastoma are planned to undergo this CAR-T cell treatment [59]. These encouraging findings accentuate the effectiveness of humanized CAR-T cells for the treatment of patients who did not achieve remission or grew resistant to murine- or camelid-based CAR-T cells due to the immunogenicity of the antigen-recognition domains [49, 55, 56].

Other researchers have also humanized VHHs and have reported that mutation of residues in frameworks other than frameworks 2 does not negatively impact VHH properties [60]. According to an investigation, Vincke and colleagues also reported that the humanization of some residues (namely, Glu49 and Arg50 to Gly and Leu, respectively) would contribute to increasing the stability of the humanized VHH despite lowering its solubility index [60]. These researchers also identified two framework 2 residues (namely, Phe42 and Gly/Ala52 to Val and Trp, respectively) whose substitutions would result in the loss of antigen affinity of the VHH [60]. Ultimately, Vincke and colleagues introduced a universal VHH scaffold that could be leveraged for the humanization of any given VHH without the loss of antigen affinity or specificity [60]. However, in this study, our strategy of humanization was a rather different one, and H85 did not undergo any residue substitution in framework 2 following humanization. In 2023, Temple and colleagues humanized a llama-derived CD72-specific VHH (named NbD4) and demonstrated that the humanized version of the VHH (named H24) exhibits a higher affinity to CD72 in comparison with the native VHH [61]. Furthermore, Temple and colleagues asserted that framework residue substitution through humanization could be a potential strategy for maximizing the antitumor efficacy of VHH-based CAR-T cells, as was the case for H24-based CD72-redirected CAR-T cells [61].

It has been recently evident that the presence of different CD19 isoforms contributes to the emergence of resistance to conventional CD19-redirected CAR-T cell therapies since the currently used antigen-recognition domains fail to recognize or bind these isoforms [8, 62]. This occurrence further highlights the necessity of developing CAR-T cells that are equipped with different antigen-recognition domains, rather than the murine CD19-specific scFv FMC63, that can target CD19 epitopes different from those targeted by conventional CD19-redirected CAR T cells [62]. In this regard, Gu and colleagues used the anti-CD19 scFv HI19α for the generation of a CD19-redirected CAR-T cell product, named CNCT19, which was later investigated in a clinical trial (NCT02975687) for the treatment of twenty individuals with R/R B-ALL [62]. Briefly, these researchers reported CR/CRi in 90% of the treated patients in less than a month, which highlights the clinical applicability of CNCT19 for the treatment of CD19-associated malignancies [62]. Such findings support the fact that there is an urgent need for the development of novel CD19-redirected CAR-T cell products whose antigen-recognition domains are composed of novel targeting moieties. Additionally, another potent strategy for tackling the resistance to conventional CD19-redirected CAR-T cells can be based upon the selection of more than one CD19-specific antigen-recognition domain (two or three distinct CD19-specific VHHs, such as those previously isolated in our lab) [10]. Such VHHs can be employed for the development of oligoclonal antigen-recognition domains since the combination of oligoclonal VHH with third-generation CARs can substantially improve the tumoricidal functionality of CAR-T cells and enable them to simultaneously target multiple CD19 epitopes [63]. In this regard, Jamnani and colleagues have previously demonstrated that HER2-redirected CAR-T cells with oligoclonal VHH-based antigen-recognition domains are capable of higher proliferation and proinflammatory cytokine production alongside mediating more pronounced antitumor reactions as compared with their non-oligoclonal counterparts [63].

In silico techniques, such as those employed in this study, could be considered an established method for the humanization of targeting moieties, especially VHHs. Moreover, such techniques could also be utilized for adjusting the affinity of any given antigen-recognition domain to its corresponding antigen since it has been demonstrated that CAR-T cells with affinity-tuned targeting domains are able to discriminate between healthy cells that express the antigen at physiologic levels and tumor cells that overexpress it [64]. Since commonly targeted antigens are usually expressed by both healthy and malignant cells, CAR-T cell-mediated antitumor responses lead to the elimination of healthy cells as well, creating a phenomenon referred to as “*on-target/off-tumor*” toxicities [65]. In the case of CD19-redirected CAR-T cell therapies, such toxicities result in the elimination of normal B cells, commonly referred to as B-cell aplasia, which consequently places the patients in need of immunoglobulin replacement to circumvent opportunistic infections [65]. In this regard, it has been demonstrated that reducing or adjusting the affinity of the antigen-recognition domains of CAR constructs can result in sufficient tumoricidal efficacy of CAR-T cells, similar to those of high-affinity CAR-T cells, and enable them to spare healthy cells, thus minimizing the unfavorable damages and maximizing the applicability of CAR-T cell therapies [64].

The current study lays the groundwork for meticulous in silico antibody engineering techniques that offer a great deal of flexibility for the engineering of desired targeting moieties that alongside their potential application as the antigen-recognition domains of CAR-T cells have various other potentials which include the development of ADCs or bispecific/bivalent biparatopic VHHs for various types of immunotherapies.

## Conclusion

CD19-redirected CAR-T cells have been approved as the second-line and third-line treatments for certain patients with CD19+ leukemias and lymphomas. However, the immunogenicity of their antigen-recognition domains might trigger host immune responses that incapacitate the antitumor reactivity of the infused CAR-T cells. Humanization of such antigen-recognition domains might be a potential strategy for minimizing such anaphylactic events and maximizing the therapeutic benefit of CAR-T cells. Herein, a CD19-specific VHH, H85, was humanized using in-depth and precise in silico techniques, and HuH85CAR-T cells demonstrated CAR expression and surface density rates, cytokine secretion profiles, and antitumor effects comparable to those of H85CAR-T cells. Future studies should focus on the characterization and comparison of HuH85CAR-T cells with H85CAR-T cells in terms of their proliferation capacity, phenotypic characteristics, and antitumor effects in xenograft models of CD19-associated malignancies. Ultimately, the assessment of the safety index and therapeutic efficacy of HuH85CAR-T cells warrants clinical investigation.

### Electronic supplementary material

Below is the link to the electronic supplementary material.

Supplementary Material 1

## Data Availability

The datasets used and/or analysed during the current study are available from the corresponding author on reasonable request.

## Abbreviations

mAbs: Monoclonal antibodies
TRBAs: T-cell-redirecting bispecific antibodies
ADCs: Antibody-drug conjugates
CAR: Chimeric antigen receptor
scFv: Single-chain fragment variable
MHC: Major histocompatibility complex
FDA: Food and Drug Administration
R/R: Relapsed/refractory
B-ALL: B-cell acute lymphoblastic leukemia
DLBCL: Diffuse large B-cell lymphoma
MCL: Mantle cell lymphoma
V_H_: Hypervariable region of the heavy chain
PDB: Protein Data Bank
CDRs: Complementarity-determining regions
RMSD: Root-mean-square deviation
IEDB: Immune Epitope Data Base
MD: Molecular Dynamics
ps: Picoseconds
R_g_: Radius of gyration
ns: Nanoseconds
K_d_: Dissociation constant
HEK 293LTV: Human embryonic kidney 293LTV
DMEM: Dulbecco’s Modified Eagles Medium
FBS: Fetal bovine serum
PEI: Polyethyleneimine
RPMI 1640: Roswell Park Memorial Institute 1640
qPCR: Quantitative real-time PCR
PBS: Phosphate-buffered saline
PMBC: Peripheral blood mononuclear cells
IL-2: Interleukin 2
MOI: Multiplicity of infection
MFI: Mean fluorescence intensity
LDH: Lactate dehydrogenase
E:T: Effector to target
RMSF: Root-mean-square fluctuation
FL: Follicular lymphoma
CR: Complete remission
CRi: CR with incomplete count recovery
