# Supplementary material for: Humanization of the antigen-recognition domain does not impinge on the antigen-binding, cytokine secretion, and antitumor reactivity of humanized nanobody-based CD19-redirected CAR-T cells
Source: J Transl Med. 2024 Jul 25;22:679. doi: 10.1186/s12967-024-05461-8 (PMC11271212; doi:10.1186/s12967-024-05461-8)
Supplement: Supplementary file 1 — Supplementary Material 1 [file 12967_2024_5461_MOESM1_ESM.docx]

**Supplemental Table 1** The results of the meticulous structural assessments of each of the predicted 3D models using QMEANDisCo, ProSA, and MolProbity

| **Nanobody** | **The best 3D model of each server used for structure prediction** | **QMEANDisCo** | **ProSa (Z-Score)** | **Ramachandran analysis** | | | | **Further Geometry analyses** | | | | | | **Peptide Omegas** | | **Low-resolution Criteria** | | **All-Atom Contacts** |  |
| --- | --- | --- | --- | --- | --- | --- | --- | --- | --- | --- | --- | --- | --- | --- | --- | --- | --- | --- | --- |
|  |  |  |  | Residues in favored regions  (>98%) | Residues in allowed regions (>99.8%) | Outliers (<0.05%) | Rama distribution Z-score (abs(Z score) < 2) | Bad bonds (Goal: 0%) | | Bad angles (Goal: <0.1%) | Poor rotamers (Goal: <0.3%) | Favored rotamers (Goal: >98%) | Cβ deviations >0.25Å (Goal: 0) | Cis Prolines (Expected: ≤1 per chain, or ≤5%) | Twisted Peptides (Goal: 0) | CaBLAM outliers (Goal: <1.0%) | CA Geometry outliers (Goal: <0.5%) | Clashscore, all atoms (percentile)* | MolProbity score (percentile) |
| **H85** | Robetta Model 1 | 0.84 ± 0.08 | -6.24 | 98.3% (113/115) | 99.1% (114/115) | 1 (0.87%) | 0.99 ± 0.86 | 1 / 915 (0.11%) | 3 / 1239 (0.24%) | | 0 (0.00%) | 95 (100.00%) | 0 (0.00%) | 0/3 (0.00%) | 0 / 116 (0.00%) | 2 (1.8%) | 0 (0.00%) | 1.71 (99^th^) | 0.92 (100^th^) |
|  | GalaxyWEB Model 1 | 0.86 ± 0.08 | -6.15 | 98.3% (113/115) | 99.1% (114/115) | 1 (0.87%) | 0.22 ± 0.77 | 4 / 915 (0.44%) | 9 / 1239 (0.73%) | | 1 (1.05%) | 92 (96.84%) | 4 (3.92%) | 0/3 (0.00%) | 0 / 116 (0.00%) | 4 (3.5%) | 0 (0.00%) | 12.52 (61^st^) | 1.63 (92^nd^) |
|  | I-TASSER Model 1 | 0.77 ± 0.08 | -6.31 | 79.1% (91/115) | 92.2% (106/115) | 9 (7.83%) | -6.48 ± 0.62 | 0 / 914 (0.00%) | 16 / 1237 (1.29%) | | 4 (4.21%) | 75 (78.95%) | 4 (3.92%) | 0/3 (0.00%) | 6 / 116 (5.17%) | 10 (8.8%) | 3 (2.65%) | 3.42 (97th) | 2.35 (56^th^) |
| **HuH85** | Robetta Model 1 | 0.85 ± 0.08 | -6.93 | 96.5% (111/115) | 99.1% (114/115) | 1 (0.87%) | 1.55 ± 0.83 | 4 / 910 (0.44%) | 7 / 1232 (0.57%) | | 0 (0.00%) | 94 (98.95%) | 0 (0.00%) | 0/2 (0.00%) | 0 / 116 (0.00%) | 3 (2.7%) | 0 (0.00%) | 1.14 (99^th^) | 1.05 (100^th^) |
|  | GalaxyWEB Model 1 | 0.84 ± 0.08 | -6.13 | 98.3% (113/115) | 100.0% (115/115) | 0 (0.00%) | -1.26 ± 0.69 | 2 / 910 (0.22%) | 12 / 1232 (0.97%) | | 0 (0.00%) | 94 (98.95%) | 2 (1.96%) | 0/2 (0.00%) | 0 / 116 (0.00%) | 1 (0.9%) | 1 (0.88%) | 13.11 (57^th^) | 1.63 (92th) |
|  | I-TASSER Model 1 | 0.79 ± 0.08 | -6.64 | 83.5% (96/115) | 96.5% (111/115) | 4 (3.48%) | -4.47 ± 0.76 | 0 / 910 (0.00%) | 12 / 1231 (0.97%) | | 8 (8.42%) | 75 (78.95%) | 4 (3.92%) | 0/2 (0.00%) | 4 / 116 (3.45%) | 3 (2.7%) | 1 (0.88%) | 3.43 (97^th^) | 2.52 (46^th^) |

*Clashscore is the number of serious steric overlaps (> 0.4 Å) per 1000 atoms (100th percentile indicates the best among structures of comparable resolution; 0th percentile denotes the worst.)
